# Supplementary material for: Pre-existing and early cellular immune factors correlate with functionally complete protection against primary controlled human SARS-CoV-2 infection
Source: Nat Commun. 2025 Dec 7;17:312. doi: 10.1038/s41467-025-67017-8 (PMC12789448; doi:10.1038/s41467-025-67017-8)
Supplement: Supplementary file 1 — Supplementary Information [file 41467_2025_67017_MOESM1_ESM.pdf]

Pre-existing and early cellular immune factors correlate with functionally complete protection against primary controlled human SARS-CoV-2 infection

Supplementary Figures 1-7 and Supplementary Tables 1-5.

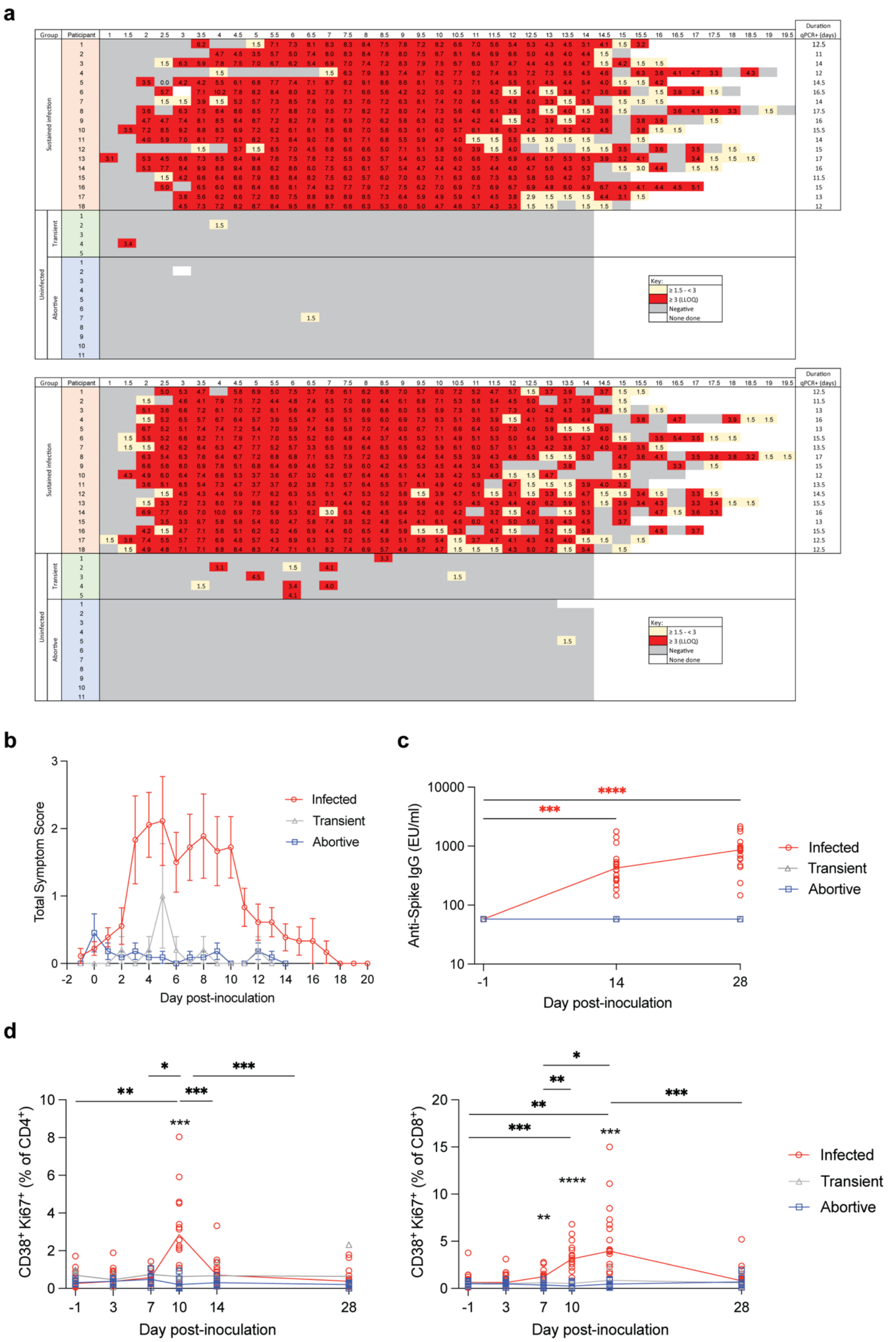

Supplementary Fig. 1. a, Viral load (nasal swab N gene copies/ml, qPCR) by participant in each of the

three outcome groups, red represents positive detections, yellow; detections below LLOQ and grey; negative. **b**, Daily total symptom scores pre- and post-inoculation. **c**, Anti-spike IgG measured by ELISA, LLOD 50.2 EU/ml and **d**, *ex vivo* $CD4^+$ and $CD8^+$ $CD38^+Ki67^+$ frequency before and after inoculation in the infected (n=18), transient (n=5) and abortive (n=11) sub-groups. Lines show mean ±SEM (b) or line at median (c, d), two-way ANOVA mixed-effects models with Geisser-Greenhouse correction and Tukey's (longitudinal) or Holm-Šídák's (between groups) multiple comparisons test. *p<0.05, **p<0.01, ***p<0.001.

a Baseline antibody; plasma

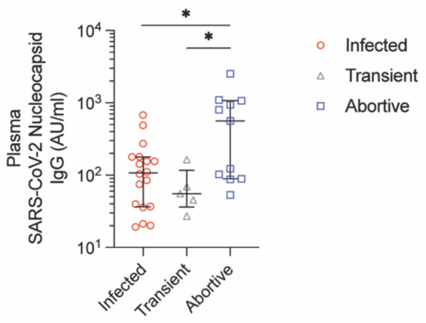

b Baseline antibody; nasal lining fluid

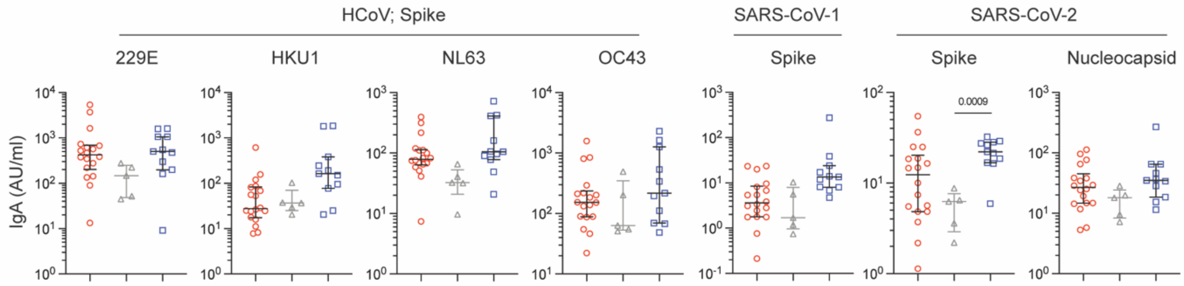

Supplementary Fig. 2. a, Plasma anti-SARS-CoV-2 nucleocapsid IgG concentration spit into the three infection outcomes; infected (n=18), transient (n=5) and abortive (n=11). Line and error show median and IQR. One-way ANOVA mixed-effects model with Šídák's multiple comparisons test (a). For correction for the number of antigens and isotypes in b, two-way ANOVA with Geisser-Greenhouse correction and Šídák's multiple comparisons test was used. *p<0.05.

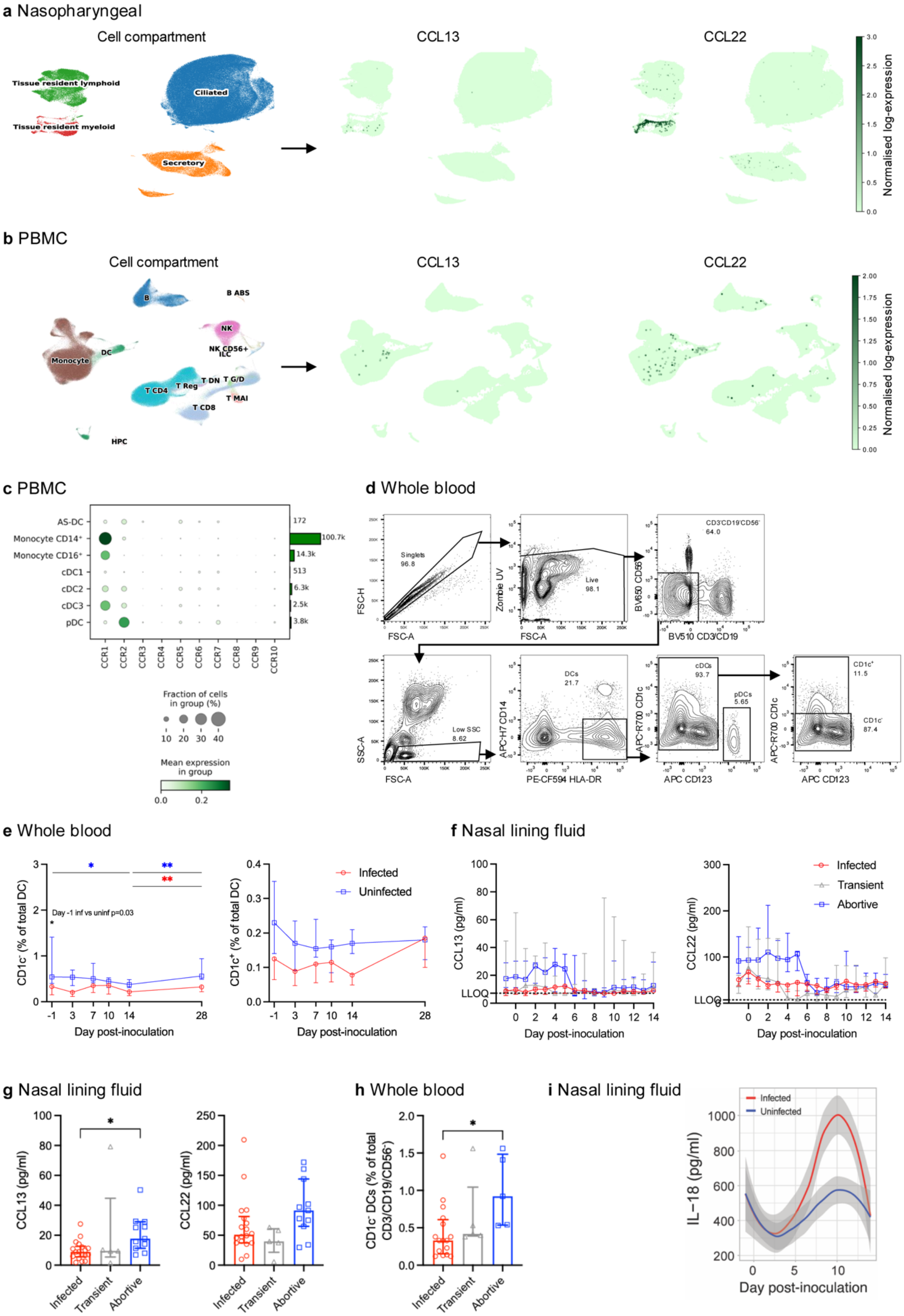
a Nasopharyngeal
Cell compartment
CCL13
CCL22
Tissue resident lymphoid
Tissue resident myeloid
Ciliated
Secretory
Normalised log-expression
b PBMC
Cell compartment
CCL13
CCL22
B
B ABS
NK
NK CD56+
ILC
Monocyte
DC
T CD4
T Reg
T DN
T G/D
T MAI
T CD8
HPC
Normalised log-expression
c PBMC
AS-DC
Monocyte CD14+
Monocyte CD16+
cDC1
cDC2
cDC3
pDC
172
100.7k
14.3k
513
6.3k
2.5k
3.8k
CCR1
CCR2
CCR3
CCR4
CCR5
CCR6
CCR7
CCR8
CCR9
CCR10
Fraction of cells in group (%)
10 20 30 40
Mean expression in group
0.0 0.2
d Whole blood
Singlets 96.8
FSC-H
FSC-A
Zombie UV
Live 98.1
CD3-CD19-CD56- 64.0
BV650 CD56
BV510 CD3/CD19
SSC-A
Low SSC 8.62
DCs 21.7
APC-H7 CD14
PE-CF594 HLA-DR
cDCs 93.7
pDCs 5.65
APC-R700 CD1c
APC CD123
CD1c+ 11.5
CD1c- 87.4
e Whole blood
CD1c- (% of total DC)
Day -1 inf vs uninf p=0.03
CD1c+ (% of total DC)
Day post-inoculation
Infected
Uninfected
f Nasal lining fluid
CCL13 (pg/ml)
CCL22 (pg/ml)
LLOQ
Day post-inoculation
Infected
Transient
Abortive
g Nasal lining fluid
CCL13 (pg/ml)
CCL22 (pg/ml)
Infected
Transient
Abortive
h Whole blood
CD1c- DCs (% of total CD3/CD19/CD56-)
Infected
Transient
Abortive
i Nasal lining fluid
IL-18 (pg/ml)
Infected
Uninfected
Day post-inoculation

Supplementary Fig. 3. **a**, Nasopharyngeal or **b**, PBMC compartments showing CCL13 and CCL22 transcript expression. **c**, PBMC myeloid cell CCR gene expression across all timepoints and infection groups. Circles represent the fraction of cells expressing CCL13 or CCL22, circle colour indicates higher expression. Bars show the number of cells sequenced. **d**, Representative gating strategy for blood DC subset analysis presented in Fig. 3, DCs were gated as single, live, $CD3^{-}CD19^{-}CD56^{-}$, low SSC, HLA-$DR^{+}CD14^{-}$. **e**, Blood $CD1c^{-}$ and $CD1c^{+}$ DC population frequencies as a percentage of the $CD3^{-}CD19^{-}CD56^{-}$ gate pre and post-inoculation in the infected (n=18) and uninfected (n=16) groups. **f**, Nasal lining fluid CCL13 and CCL22 protein concentration pre and post-inoculation in the infected (n=18), transient (n=5) and abortive (n=11) groups. **g**, Nasal lining fluid CCL13 and CCL22 protein concentration at baseline in the infected (n=18), transient (n=5) and abortive (n=11) groups. **h**, Baseline frequency of blood $CD1c^{-}$ DCs as a percentage of the $CD3^{-}CD19^{-}CD56^{-}$ gate in the infected (n=18), transient (n=5) and abortive (n=11) groups. **i**, IL-18 concentration in daily nasal lining fluid samples pre- and post-inoculation in the infected (n=18) and uninfected (n=16) groups shown as loess plot. Bar or line and error show median and IQR, two-way ANOVA mixed-effects models with Geisser-Greenhouse correction and Tukey's (longitudinal) or Holm-Šídák's (between groups) multiple comparisons test (e, f) and one-way ANOVA Kruskal-Wallis test with Dunn's multiple comparisons test (g). *p<0.05, **p<0.01.

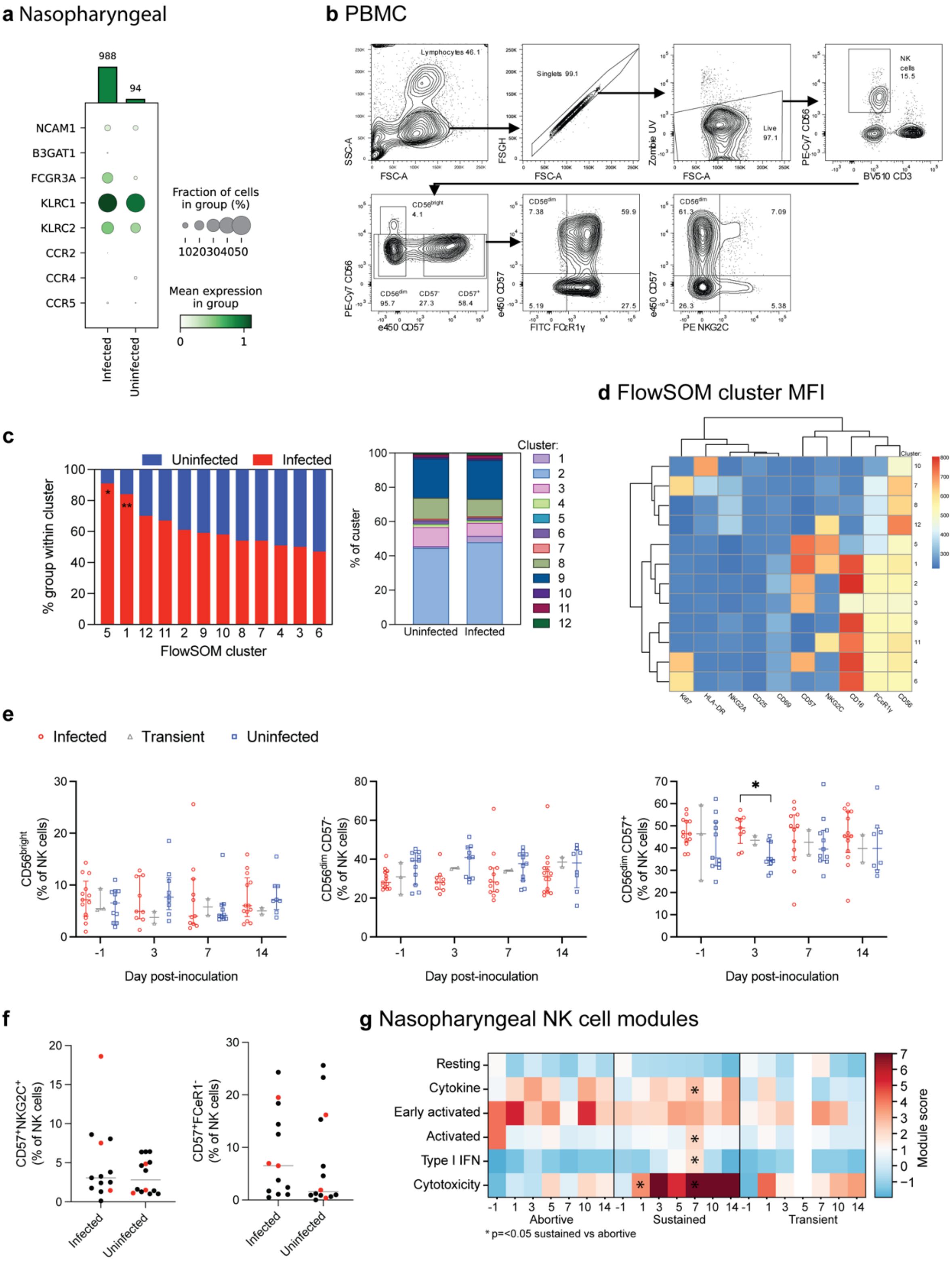

a Nasopharyngeal
988
94
NCAM1
B3GAT1
FCGR3A
KLRC1
KLRC2
CCR2
CCR4
CCR5
Fraction of cells in group (%)
1020304050
Mean expression in group
0
1
Infected
Uninfected
b PBMC
Lymphocytes 46.1
SSC-A
FSC-A
Singlets 99.1
FSC-H
Zombie UV
Live 97.1
PE-Cy7 CD56
NK cells 15.5
BV510 CD3
CD56bright 4.1
CD56dim 95.7
CD57- 27.3
CD57+ 58.4
e450 CD57
CD56dim 7.38
59.9
5.19
27.5
FITC FcεR1γ
CD56dim 61.3
7.09
26.3
5.38
PE NKG2C
c
Uninfected
Infected
% group within cluster
FlowSOM cluster
% of custer
Cluster:
d FlowSOM cluster MFI
Ki67
HLA-DR
NKG2A
CD25
CD69
CD57
NKG2C
CD16
FCεR1γ
CD56
e
Infected
Transient
Uninfected
CD56bright (% of NK cells)
CD56dim CD57- (% of NK cells)
CD56dim CD57+ (% of NK cells)
Day post-inoculation
f
CD57+NKG2C+ (% of NK cells)
CD57+FCeR1- (% of NK cells)
Infected
Uninfected
g Nasopharyngeal NK cell modules
Resting
Cytokine
Early activated
Activated
Type I IFN
Cytotoxicity
Abortive
Sustained
Transient
Module score
* p=<0.05 sustained vs abortive

Supplementary Fig. 4. a, Nasopharyngeal NK cell gene expression profile in the infected (n=6) and uninfected (n=10) groups, circle colour indicates higher expression, bars show the number of cells sequenced. **b**, Representative manual gating strategy for NK cell subsets used in Fig. 4. **c**, Frequency of group within FlowSOM clusters derived from the NK cell t-SNE map in Fig. 4a split by infected (n=13) and uninfected (n=14) groups and cluster frequency in each group. **d**, FlowSOM cluster marker MFI. **e**, NK cell subset frequencies determined by CD56 and CD57 at day -1, 3, 7 or 10 and 14 post-inoculation split by the 3 groups (baseline n=13 infected, n=3 transient, n=11 abortive, day 3 n=9, 2, 10, day 7/10 n=12, 2, 11, day 14 n=14, 2, 18, day 28 n=12, 4, 11 respectively). **f**, $CD56^{dim}CD57^{+}NKG2C^{+}$ and $CD57^{+}FC\varepsilon R1\gamma^{-}$ NK cell frequency at baseline in the infected and uninfected groups showing the participants who tested positive for HCMV serology in red. **g**, Nasopharyngeal NK cell gene expression module scores across the time course in the three groups (n=6 infected, n=3 transient, n=7 abortive). Line at median and IQR (e) or median only (f). Two-way ANOVA mixed-effects models with Geisser-Greenhouse correction and Tukey's (longitudinal) or Holm-Šídák's (between groups) multiple comparisons test (e), Mann-Whitney unpaired U test (f). *p<0.05.

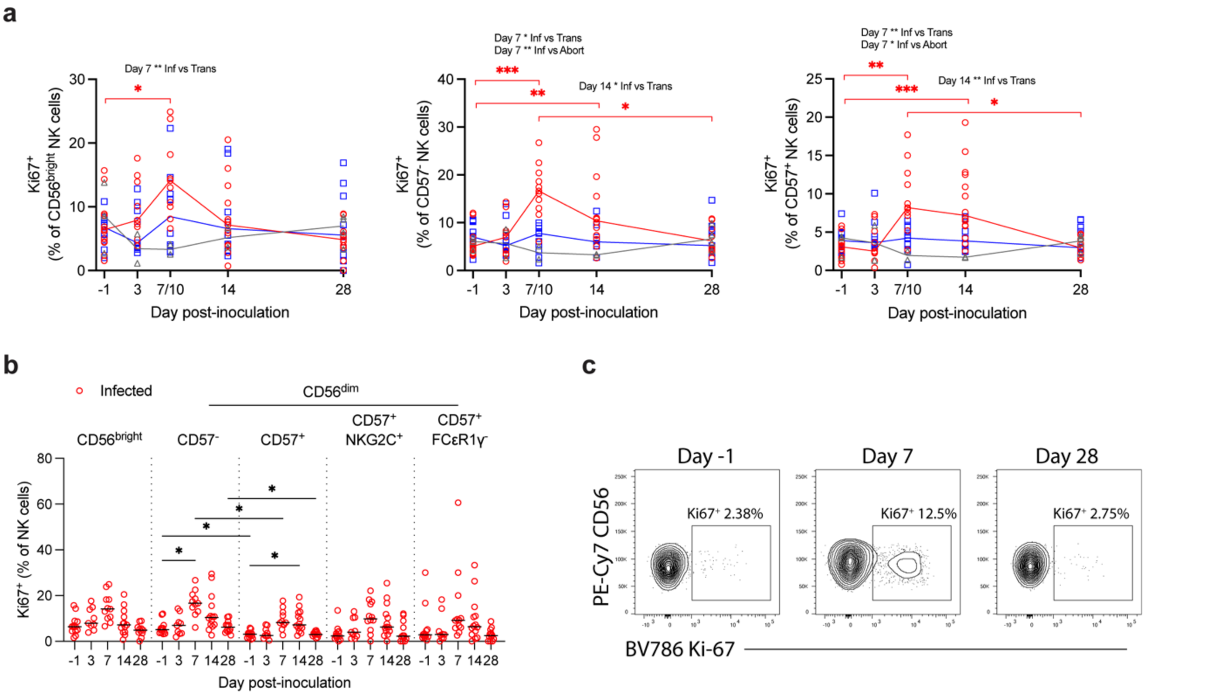

Supplementary Fig. 5. a, NK cell Ki67 expression at day -1, 3, 7 or 10, 14 and 28 post-inoculation in the 3 groups. **b**, NK cell Ki67 expression in the infected group only in subsets defined by CD56, CD57, NKG2C and FCεR1γ⁻ (baseline n=13 infected, n=3 transient, n=11 abortive, day 3 n=9, 2, 10, day 7/10 n=12, 2, 11, day 14 n=14, 2, 18, day 28 n=12, 4, 11 respectively). **c**, Representative gating strategy for NK cell Ki67 expression shown in a and Fig 4h. Line at median. Two-way ANOVA mixed-effects models with Geisser-Greenhouse correction and Tukey's (longitudinal) or Holm-Šídák's (between groups) multiple comparisons test (a). One-way ANOVA mixed-effects models with Geisser-Greenhouse correction and Holm-Šídák's multiple comparisons test (b). *p<0.05, **p<0.01, ***p<0.001.

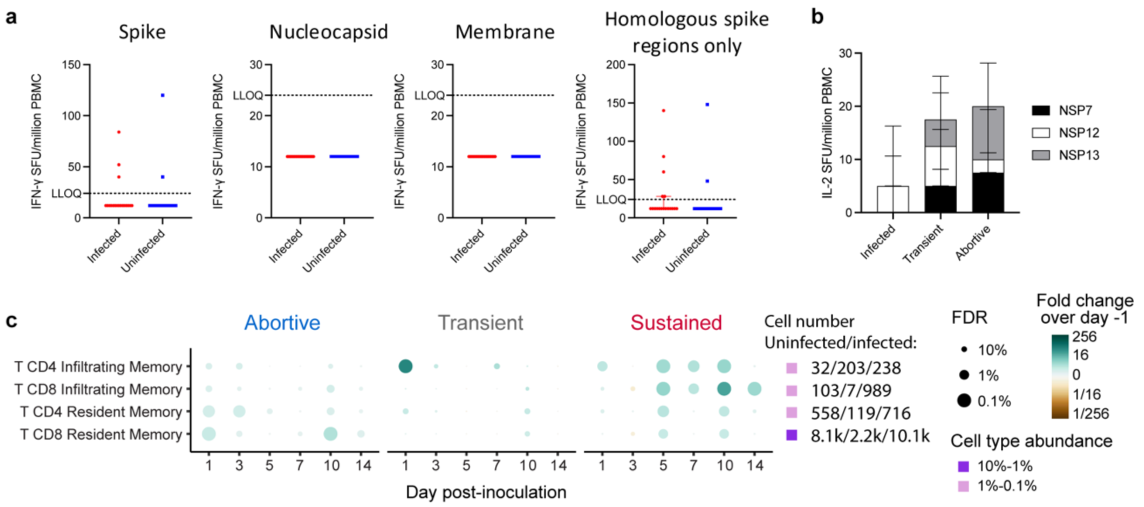

Supplementary Fig. 6. a, Baseline IFN-γ spot forming units (SFU) per million PBMC in response to spike, nucleocapsid and membrane peptide pool stimulation, or stimulation with spike peptides of homologous regions only (cross-reactive), measured by ELISpot (infected n=18, uninfected n=16). **b**, Stacked graph of baseline IL-2 SFU per million PBMC of the infected (n=13), transient (n=5) and abortive (n=10) groups measured by Fluorospot. **c**, scRNA sequencing showing abundance of infiltrating T cells and tissue resident T cells in the nasopharynx, fold change over baseline (day -1) in the infected (n=6), transient (n=3) and uninfected (n=7) groups. Line and error show median and IQR, Mann-Whitney unpaired test (a) or one-way ANOVA Kruskal-Wallis test with Dunn's multiple comparisons test (b).

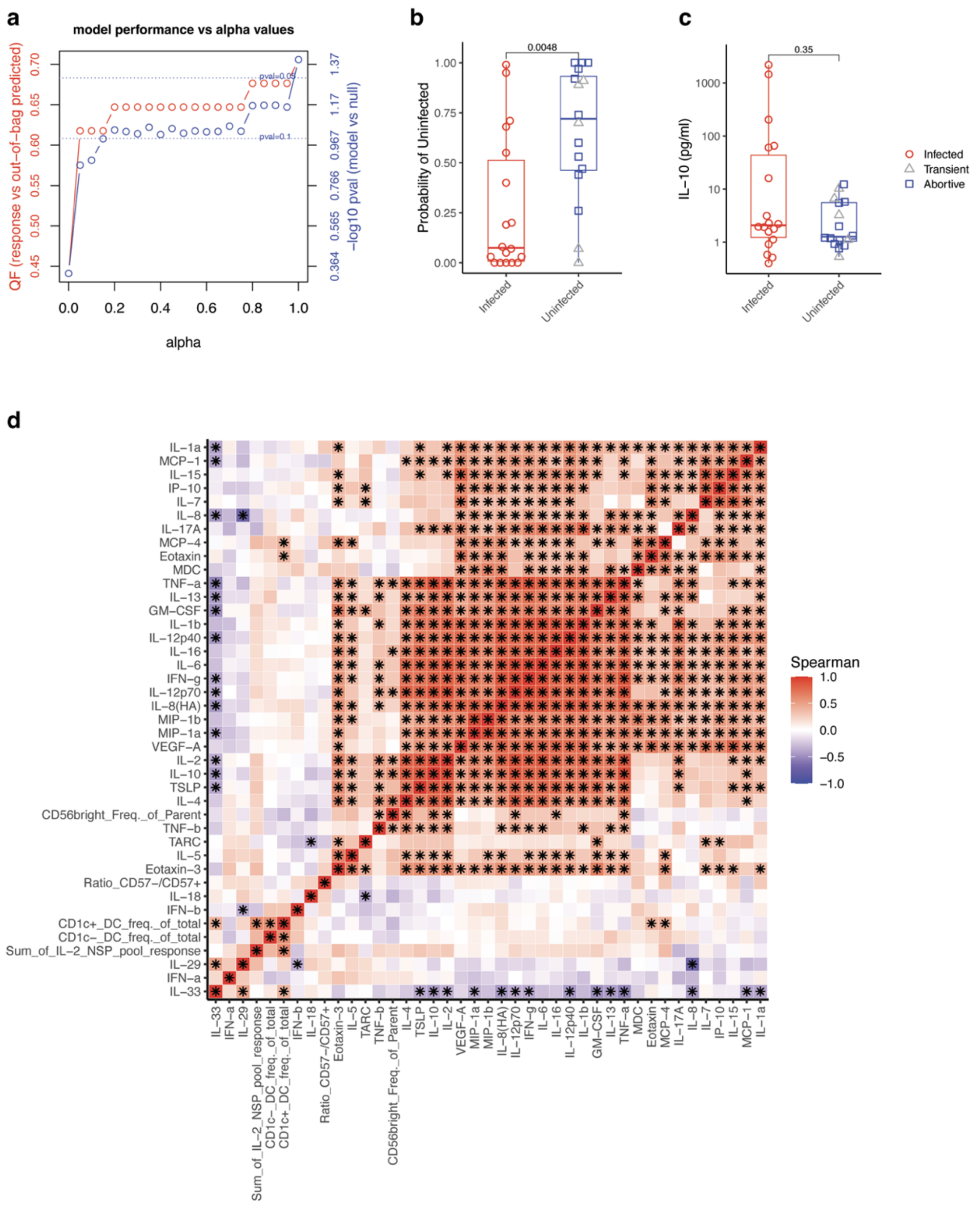

Supplementary Fig. 7. a, Elastic net model performance across alpha values, which tunes between L1 (alpha =1) and L2 (alpha = 0) regularization. Models were trained to classify the uninfected group versus the infected group using baseline nasal soluble mediators as input. Red, classification accuracy (left y-axis). Blue, model significance (-log(p-value), right y-axis) derived from null models generated from permuted samples. **b**, Probability of assignment to the uninfected group, estimated as the proportion of being predicted as uninfected across 100 cross-validation runs. Group difference was accessed using the Mann-Whitney unpaired U test. **c**, Baseline nasal lining fluid IL-10 concentrations in infected and uninfected groups measured by MSD. **d,** Pairwise Spearman correlations and corresponding p-values between baseline immune factors. Box and whisker plots show minimum to

maximum, median, 25th and 75th percentile and all points. Group difference was accessed using the Mann-Whitney unpaired U test. *$p<0.05$.

Supplementary Table 1: Comparisons of 35 soluble mediators in the nasal lining fluid between the infected and uninfected groups at baseline **day 0**. Mann-Whitney U tests without correction for multiple comparisons.

Mediator	Discover y?	P value	Mean ra nk of Inf ected	Mean ran k of Uninf ected	Mean ran k diff.	Mann-Whitney U	-log10(P v alue)
Eotaxin	No	0.153989	15.17	20.13	-4.958	102	0.8125
Eotaxin-3	No	0.38379	16.06	19.13	-3.069	118	0.4159
GM-CSF	No	0.986421	17.44	17.56	-0.1181	143	0.005938
IFNα	No	0.511105	18.58	16.28	2.302	124.5	0.2915
IFNβ	No	0.612955	16.67	18.44	-1.771	129	0.2126
IFNγ	No	0.746302	16.94	18.13	-1.181	134	0.1271
IL-10	No	0.329942	19.11	15.69	3.424	115	0.4816
IL-12p40	No	0.851471	17.83	17.13	0.7083	138	0.06983
IL-12p70	No	0.905162	17.28	17.75	-0.4722	140	0.04327
IL-13	No	0.645548	16.72	18.38	-1.653	130	0.1901
IL-15	No	0.528148	18.56	16.31	2.243	125	0.2772
IL-16	No	0.621236	18.33	16.56	1.771	129	0.2067
IL-17A	No	>0.999999	17.5	17.5	0	144	0
IL-18	Yes	**0.042465**	20.78	13.81	6.965	85	1.372
IL-1α	No	0.281118	15.72	19.5	-3.778	112	0.5511
IL-1β	No	0.645548	16.72	18.38	-1.653	130	0.1901
IL-2	No	0.42243	18.83	16	2.833	120	0.3742
IL-29	No	0.28507	19.25	15.53	3.719	112.5	0.545
IL-33	No	0.23732	19.44	15.31	4.132	109	0.6247
IL-4	No	0.824859	17.89	17.06	0.8264	137	0.08362
IL-5	No	0.778604	17.03	18.03	-1.003	135.5	0.1087
IL-6	No	0.484353	16.33	18.81	-2.479	123	0.3148
IL-7	No	0.670233	18.22	16.69	1.535	131	0.1738
IL-8	No	0.573822	16.56	18.56	-2.007	127	0.2412
CXCL10	No	0.720635	16.89	18.19	-1.299	133	0.1423
CCL2	No	0.329942	15.89	19.31	-3.424	115	0.4816
CCL13	Yes	**0.019895**	13.78	21.69	-7.91	77	1.701
CCL22	No	0.164344	15.22	20.06	-4.84	103	0.7842
CCL3	No	0.746302	16.94	18.13	-1.181	134	0.1271
CCL4	No	0.959279	17.61	17.38	0.2361	142	0.01806
TARC	No	0.819663	17.89	17.06	0.8264	137	0.08636
TNF	No	0.986421	17.44	17.56	-0.1181	143	0.005938
LTα	No	0.81008	17.11	17.94	-0.8264	137	0.09147
TSLP	No	0.186568	19.67	15.06	4.604	105	0.7292
VEGF-A	No	0.959279	17.39	17.63	-0.2361	142	0.01806

Supplementary Table 2: Comparison of CCL13 levels between the infected and uninfected groups by day post-inoculation. Mann-Whitney U tests with P-value adjustment using the Holm-Šídák method.

Day post-inoculation	Below threshold?	P value	Mean rank of Infected	Mean rank of Uninfected	Mean rank diff.	Mann-Whitney U	Adjusted P Value
-1	No	0.020826	13.81	21.66	-7.851	77.5	0.260243
0	No	0.019895	13.78	21.69	-7.91	77	0.260243
1	No	0.006315	13.17	22.38	-9.208	66	0.096394
2	Yes	**0.001592**	12.56	23.06	-10.51	55	0.026715
3	No	0.198455	15.39	19.88	-4.486	106	0.929671
4	No	0.232627	15.56	19.69	-4.132	109	0.929671
5	No	0.314	15.86	19.34	-3.483	114.5	0.950955
6	No	0.486947	18.64	16.22	2.42	123.5	0.981753
7	No	0.574914	18.42	16.47	1.948	127.5	0.981753
8	No	0.602325	18.36	16.53	1.83	128.5	0.981753
9	No	0.897821	17.28	17.75	-0.4722	140	0.981753
10	No	0.033866	14.11	21.31	-7.201	83	0.361021
11	No	0.486902	16.36	18.78	-2.42	123.5	0.981753
12	No	0.206499	15.44	19.81	-4.368	107	0.929671
13	No	0.377851	16.06	19.13	-3.069	118	0.96392
14	No	0.243455	15.17	19.2	-4.033	102	0.929671
28	No	0.598227	16.17	18	-1.833	120	0.981753

Supplementary Table 3: Comparisons of 35 soluble mediators in the nasal lining fluid between the infected and uninfected groups at baseline **day -1**. Mann-Whitney U tests without correction for multiple comparisons.

Mediator	Discover y?	P value	Mean ran k of Infec ted	Mean ran k of Unin fected	Mean ran k diff.	Mann-Whitney U	-log10(P v alue)
Eotaxin	No	0.059393	20.56	14.06	6.493	89	1.226
Eotaxin-3	No	0.490005	18.64	16.22	2.42	123.5	0.3098
GM-CSF	No	0.986421	17.44	17.56	-0.1181	143	0.005938
IFNα	No	0.296107	19.22	15.56	3.66	113	0.5286
IFNβ	No	0.695178	18.14	16.78	1.358	132.5	0.1579
IFNγ	No	0.824859	17.89	17.06	0.8264	137	0.08362
IL-10	No	0.296832	15.78	19.44	-3.66	113	0.5275
IL-12p40	No	0.959279	17.61	17.38	0.2361	142	0.01806
IL-12p70	No	0.746302	18.06	16.88	1.181	134	0.1271
IL-13	No	0.550758	18.5	16.38	2.125	126	0.259
IL-15	No	0.210872	19.56	15.19	4.368	107	0.676
IL-16	No	0.645548	16.72	18.38	-1.653	130	0.1901
IL-17A	No	0.851471	17.17	17.88	-0.7083	138	0.06983
IL-18	No	0.573822	18.44	16.44	2.007	127	0.2412
IL-1α	No	0.125817	20	14.69	5.313	99	0.9003
IL-1β	No	0.824859	17.11	17.94	-0.8264	137	0.08362
IL-2	No	0.824859	17.11	17.94	-0.8264	137	0.08362
IL-29	No	0.597321	18.39	16.5	1.889	128	0.2238
IL-33	No	0.720635	18.11	16.81	1.299	133	0.1423
IL-4	No	0.932182	17.67	17.31	0.3542	141	0.0305
IL-5	No	0.360819	16	19.19	-3.188	117	0.4427
IL-6	No	0.932182	17.67	17.31	0.3542	141	0.0305
IL-7	No	0.251365	19.39	15.38	4.014	110	0.5997
IL-8	No	0.932182	17.67	17.31	0.3542	141	0.0305
CXCL10	No	0.621236	18.33	16.56	1.771	129	0.2067
CCL2	No	0.573822	18.44	16.44	2.007	127	0.2412
CCL13	No	0.060609	20.53	14.09	6.434	89.5	1.217
CCL22	No	0.511731	18.58	16.28	2.302	124.5	0.291
CCL3	No	0.720635	16.89	18.19	-1.299	133	0.1423
CCL4	No	0.506008	16.39	18.75	-2.361	124	0.2958
TARC	No	0.986012	17.44	17.56	-0.1181	143	0.006118
TNF	No	0.772247	17	18.06	-1.063	135	0.1122
LTα	No	0.814729	17.89	17.06	0.8264	137	0.08899
TSLP	No	0.281118	15.72	19.5	-3.778	112	0.5511
VEGF-A	No	0.40284	18.89	15.94	2.951	119	0.3949

Supplementary Table 4: Genes used to distinguish NK cell modules in Supplementary Fig. 4g.

Gene	Module
FCGR3A	Resting
LTB	
IFNG	Cytokine
TNF	
CCL3	
CCL4	
CSF2	
CD69	Early activated
FOS	
JUN	
EGR1	
NR4A2	
NFKBIA	
NFKBID	
NFKBIZ	
GZMB	Activated
SERPINB9	
XCL1	
XCL2	
REL	
BIRC3	
TNFRSF9	
CRTAM	
TNFRSF18	
LAG3	
ISG15	Type I IFN
ISG20	
MX1	
IFIT1	
IFIT3	
GZMA	Cytotoxicity
GZMB	
PRF1	
GNLY	
NKG7	

Supplementary Table 5: Flow cytometry antibody details.

Antibody	Clone	Catalogue number	Supplier	Dilution used
CD123 APC	6H6	306012	Biolegend	40
CD56 BV510	HCD56	318344	Biolegend	40
CD14 APC-H7	MφP9	560180	BD Biosciences	40
CD19 BV510	SJ25C1	562947	BD Biosciences	40
HLA-DR PE-CF594	G46-6	562304	BD Biosciences	40
CD1c APC-R700	F10/21A3	566614	BD Biosciences	40
FCεR1γ FITC	-	FCABS400F	Merck	20
CD25 PerCP Cy5.5	BC96	45-0259-42	Invitrogen	20
CD57 eFluor 450	TB01	48-0577-42	Invitrogen	20
NKG2A APC	S19004C	375108	Biolegend	200
CD3 BV510	UCHT1	300448	Biolegend	20
CD16 BV605	3G8	302040	Biolegend	40
CD69 BV711	FN50	310944	Biolegend	10
Viability Zombie	-	423108	Biolegend	250
NKG2C PE	S19005E	375004	Biolegend	20
KI-67 BV786	B56	563756	BD Biosciences	40
CD56 PE Cy-7	NCAM16.2	335826	BD Biosciences	40
HLA-DR APC R700	G46-6	565127	BD Biosciences	20
