# Supplementary material for: Pre-existing and early cellular immune factors correlate with functionally complete protection against primary controlled human SARS-CoV-2 infection
Source: Nat Commun. 2025 Dec 7;17:312. doi: 10.1038/s41467-025-67017-8 (PMC12789448; doi:10.1038/s41467-025-67017-8)
Supplement: Supplementary file 2 — Reporting Summary [file 41467_2025_67017_MOESM2_ESM.pdf]

nature portfolio

Corresponding author(s): Christopher Chiu

Last updated by author(s): Sep 23, 2025

Reporting Summary

Nature Portfolio wishes to improve the reproducibility of the work that we publish. This form provides structure for consistency and transparency in reporting. For further information on Nature Portfolio policies, see our Editorial Policies and the Editorial Policy Checklist.

Statistics

For all statistical analyses, confirm that the following items are present in the figure legend, table legend, main text, or Methods section.

n/a	Confirmed	
☐	☒	The exact sample size (*n*) for each experimental group/condition, given as a discrete number and unit of measurement
☐	☒	A statement on whether measurements were taken from distinct samples or whether the same sample was measured repeatedly
☐	☒	The statistical test(s) used AND whether they are one- or two-sided<br>*Only common tests should be described solely by name; describe more complex techniques in the Methods section.*
☒	☐	A description of all covariates tested
☐	☒	A description of any assumptions or corrections, such as tests of normality and adjustment for multiple comparisons
☐	☒	A full description of the statistical parameters including central tendency (e.g. means) or other basic estimates (e.g. regression coefficient) AND variation (e.g. standard deviation) or associated estimates of uncertainty (e.g. confidence intervals)
☐	☒	For null hypothesis testing, the test statistic (e.g. *F*, *t*, *r*) with confidence intervals, effect sizes, degrees of freedom and *P* value noted<br>*Give P values as exact values whenever suitable.*
☒	☐	For Bayesian analysis, information on the choice of priors and Markov chain Monte Carlo settings
☒	☐	For hierarchical and complex designs, identification of the appropriate level for tests and full reporting of outcomes
☐	☒	Estimates of effect sizes (e.g. Cohen's *d*, Pearson's *r*), indicating how they were calculated

Our web collection on statistics for biologists contains articles on many of the points above.

Software and code

Policy information about availability of computer code

Data collection	See Lindeboom et al. Nature 2024 for single cell RNA seq data collection information.
Data analysis	Graphpad Prism v10.4.1, FlowJo v10.10, R ggplot, ggplot2, tidyr, ggraph, and ggpubr. See Lindeboom et al. Nature 2024 for single cell RNA seq data analysis information.

For manuscripts utilizing custom algorithms or software that are central to the research but not yet described in published literature, software must be made available to editors and reviewers. We strongly encourage code deposition in a community repository (e.g. GitHub). See the Nature Portfolio guidelines for submitting code & software for further information.

Data

Policy information about availability of data

All manuscripts must include a data availability statement. This statement should provide the following information, where applicable:

- Accession codes, unique identifiers, or web links for publicly available datasets
- A description of any restrictions on data availability
- For clinical datasets or third party data, please ensure that the statement adheres to our policy

All data is available on request. The transcriptomic data is accessible through the COVID-19 Cell Atlas web portal: https://covid19cellatlas.org; raw sequencing data deposited under controlled access at the European Genome-Phenome Archive under accession number EGAD00001012227. The cell state annotation model is available in the CellTypist model repository (https://www.celltypist.org/models) under the name 'COVID19_HumanChallenge_Blood'. A reference for our Multi Task Gaussian Process Regression model to infer time since viral exposure on PBMC data is available at our GitHub repository (https://

github.com/Teichlab/COVID-19_Challenge_Study). Processed bulk RNAseq data is available at ArrayExpress (accession number: E-MTAB-12993). Single-cell count matrices with metadata are available at https://www.covid19cellatlas.org/ as h5ad files.

Research involving human participants, their data, or biological material

Policy information about studies with human participants or human data. See also policy information about sex, gender (identity/presentation), and sexual orientation and race, ethnicity and racism.

Reporting on sex and gender	Self-reported sex information is reported in a previous publication (Killingley et al, Nat, Med. 2022). Disaggregate sex and gender data was collected however participant numbers were too small for sex and/or gender based analysis.
Reporting on race, ethnicity, or other socially relevant groupings	Self reported race information is reported in a previous publication (Killingley et al, Nat, Med. 2022). No analysis was performed based on race.
Population characteristics	Sero suitable healthy male and female volunteers aged 18-30 years with no known risk factors for sever COVID-19 were recruited.
Recruitment	Screening of potential participants took place over two stages, initial screening visit, then a study specific remote consultation to go through study PIS and ICF. Screening took place between day -90 to day -2. Screening ICF and advertising materal were approved by the REC and HRA.<br><br>Recruitment was done under a number of channels, approved advertising, including social media. hVIVO volunteer database (volunteers registered with any another hVIVO database may be contacted to determine their interest in participating in SARS-CoV-2 research), referral and organic search (e.g. google).<br><br>The participant sample was biased by the age criteria and requirement to be healthy with no co-morbidities or known risk factors for severe COVID-19 based on clinical history, blood tests and radiology. There was potential for self-selection bias as participation was voluntary and instigated by the volunteers. Due to these factors, direct extrapolation of the results to children, older adults, thoes with pre-existing conditions and mionority groups may not be possible.
Ethics oversight	This study was conducted in accordance with the protocol, the consensus ethical principles derived from international guidlines including the Declaration of Helsinki and Council for International Organizations or Medical Sciences (CIOMS) Internaltional Ethical Guidlines, applicable ICH Good CLinical Practice guidlines, applicable laws and regulations. The screening protocol and main study were approved by the UK Health Research Authority - Ad Hoc Specialist Ethics Committee (referece: 20/UK/2001 and 20/UK/0002).

Note that full information on the approval of the study protocol must also be provided in the manuscript.

Field-specific reporting

Please select the one below that is the best fit for your research. If you are not sure, read the appropriate sections before making your selection.

[x] Life sciences   [ ] Behavioural & social sciences   [ ] Ecological, evolutionary & environmental sciences

For a reference copy of the document with all sections, see nature.com/documents/nr-reporting-summary-flat.pdf

Life sciences study design

All studies must disclose on these points even when the disclosure is negative.

Sample size	No formal sample size calculation was performed as this is and early-stage dose finding study where the primary objective was to identify a safe and infectious dose of the wild type SARS-CoV-2 virus in healthy volunteers, suitable for future intervention studies.
Data exclusions	Flow cytometry data were excluded if insufficient number of target cell type (over 50 events in the parent lineage gate), or did not pass QC (such as missing antibody), approx 4% of samples accross all data sets. Fluorospot data were excluded if there were no IFNg and/or TNFa spots in either positive controls used (FEC and ConA), 5% of samples.
Replication	The study took place over 5 distinct quarantine groups with similar findings across all.
Randomization	None, participants were enrolled into the study chronologically as they completed screening and according to their availability for at least 2 weeks quarantine. All participants were inoculated with challenge virus.
Blinding	Study particpants and participant-facing staff conducting the clinical study were blinded to the infection status where possible untill protocol-defined procedures made this apparent. Study participants and clinical staff could not be blinded to the clinical data and symptom scores as they were being generated. Laboratory staff processing the samples were blinded to infection status. Laboratory staff performing assays (qPCR, FFA, flow cytometry, fluorospot, antibody measuremenrts) were not blinded to infection status.

Reporting for specific materials, systems and methods

We require information from authors about some types of materials, experimental systems and methods used in many studies. Here, indicate whether each material, system or method listed is relevant to your study. If you are not sure if a list item applies to your research, read the appropriate section before selecting a response.

Materials & experimental systems

n/a	Involved in the study	
☐	☒	Antibodies
☐	☒	Eukaryotic cell lines
☒	☐	Palaeontology and archaeology
☒	☐	Animals and other organisms
☐	☒	Clinical data
☒	☐	Dual use research of concern
☒	☐	Plants

Methods

n/a	Involved in the study	
☒	☐	ChIP-seq
☐	☒	Flow cytometry
☒	☐	MRI-based neuroimaging

Antibodies

Antibodies used

For CITE-seq:
137 TotalSeq-C Human Cocktail, V1.0 antibodies (BioLegend, cat. # 99814399905). The reagents that were provided were a pre-diluted commercial panel.

For flow cytometry:
FceR1γ FITC FCABS400F Merck
CD57 e450 TB01 48-0577-42 Invitrogen
NKG2A APC S19004C 375108 Biolegend
CD3 V510 UCHT1 300448 Biolegend
CD16 BV605 3G8 302040 Biolegend
Viability Zombie 423108 Biolegend
NKG2C PE S19005E 375004 Biolegend
KI-67 BV786 B56 563756 BD
CD56 PE Cy-7 NCAM16.2 335826 BD
CD123 APC 6H6 306012 Biolegend
CD1c APC-R700 F10/21A3 566614 BD
CD14 APC-H7 MϕP9 560180 BD
CD19 BV510 SJ25C1 562947 BD
CD56 BV650 HCD56 318344 Biolegend
CD15 BV711 W6D3 323050 Biolegend
HLA-DR PE-CF594 G46-6 562304 BD

Validation

All antibodies employed were commercial antibodies.

Eukaryotic cell lines

Policy information about cell lines and Sex and Gender in Research

Cell line source(s)	Vero (WHO) cells purchased from the European collection of authenticated cell cultures (ECACC, cat no. 88020401)
Authentication	STR profile analysis
Mycoplasma contamination	Tested negative by culture isolation, Hoechst DNA staining and PCR
Commonly misidentified lines (See ICLAC register)	None

Clinical data

Policy information about clinical studies

All manuscripts should comply with the ICMJE guidelines for publication of clinical research and a completed CONSORT checklist must be included with all submissions.

Clinical trial registration	Clinicaltrials.gov NCT04865237
Study protocol	Available from Killingley et al. Nat Med 2022
Data collection	Conducted at the Queen Mary BioEnterprises Inovation Centre, London, UK and the Royal Free London NHS Trust, London, UK. 6th March 2021-8th July 2021
Outcomes	Primary: To identify a safe and infectious dose of wild type SARS-CoV- 2 in healthy volunteers, suitable for future intervention studies. To evaluate the safety of wild type SARS-CoV-2 challenge in healthy participants by assessing the occurrence of unsolicited AEs /SAEs

related to viral challenge.

Secondary: To further assess SARS-CoV-2 viral infection rates in upper respiratory samples in healthy volunteers, by inoculum dose. To assess the incidence of symptomatic SARS-CoV-2 infection, in healthy volunteers, by inoculum dose. To assess the SARS-CoV-2 viral dynamics (VL-AUC) in upper respiratory samples in healthy volunteers, by inoculum dose. To assess the SARS-CoV-2 induced symptoms, in healthy volunteers, by inoculum dose according to the sum total symptoms diary card score. To assess the incidence of SARS-CoV-2 illness, in healthy volunteers, by inoculum dose [Time Frame: Day 0 to discharge from Quarantine. To assess the SARS-CoV-2 viral dynamics (Peak viral load) in upper respiratory samples in healthy volunteers, by inoculum dose. To assess the SARS-CoV-2 viral dynamics (duration) in upper respiratory samples in healthy volunteers, by inoculum dose. To assess the SARS-CoV-2 viral dynamics (Incubation period) in upper respiratory samples in healthy volunteers, by inoculum dose. To assess the SARS-CoV-2 induced symptoms, in healthy volunteers, by inoculum dose according to area under the curve over time (TSS-AUC) of total clinical symptoms (TSS). To assess the SARS-CoV-2 induced symptoms, in healthy volunteers, by inoculum dose according to peak symptom diary card scores. To assess the SARS-CoV-2 induced symptoms, in healthy volunteers, by inoculum dose according to peak daily symptom score.To assess the SARS-CoV-2 induced symptoms, in healthy volunteers, by inoculum dose according to Number (%) of participants with Grade 2 or higher symptoms.

Exploratory: To explore the safety of wild type SARS-CoV-2 human challenge model in healthy adults through changes in smell (anosmia/parosmia) through infection. To explore the SARS-CoV-2 viral infection rates in saliva in healthy volunteers, by inoculum dose. To explore the SARS-CoV-2 viral dynamics in saliva in healthy volunteers, by inoculum dose. To explore the host-pathogen relationship in the SARS-CoV-2 human challenge model in healthy adults. To explore the Minimal Clinically Important Difference (MCID) in instrument-assessed change. To explore environmental contamination in the SARS-CoV-2 human challenge model in healthy adults by environmental sampling. To explore the safety of wild type SARS-CoV-2 human challenge model in healthy adults through changes in cognition through infection.To explore the safety of wild type SARS-CoV-2 human challenge model in healthy adults by assessing pulmonary changes due to experimental infection, as measured by high-resolution CT. To explore the safety of wild type SARS-CoV-2 human challenge model in healthy adults by assessing pulmonary changes due to experimental infection, as measured by Spirometry.

Plants

Seed stocks

Report on the source of all seed stocks or other plant material used. If applicable, state the seed stock centre and catalogue number. If plant specimens were collected from the field, describe the collection location, date and sampling procedures.

Novel plant genotypes

Describe the methods by which all novel plant genotypes were produced. This includes those generated by transgenic approaches, gene editing, chemical/radiation-based mutagenesis and hybridization. For transgenic lines, describe the transformation method, the number of independent lines analyzed and the generation upon which experiments were performed. For gene-edited lines, describe the editor used, the endogenous sequence targeted for editing, the targeting guide RNA sequence (if applicable) and how the editor was applied.

Authentication

Describe any authentication procedures for each seed stock used or novel genotype generated. Describe any experiments used to assess the effect of a mutation and, where applicable, how potential secondary effects (e.g. second site T-DNA insertions, mosiacism, off-target gene editing) were examined.

Flow Cytometry

Plots

Confirm that:

- [x] The axis labels state the marker and fluorochrome used (e.g. CD4-FITC).
- [x] The axis scales are clearly visible. Include numbers along axes only for bottom left plot of group (a 'group' is an analysis of identical markers).
- [x] All plots are contour plots with outliers or pseudocolor plots.
- [x] A numerical value for number of cells or percentage (with statistics) is provided.

Methodology

Sample preparation

Fresh whole blood was RBC lysed using Pharmlyse (BDbiosciences), washed in PBS then stained Zombie UV Fixable Viability Kit (Biolegend) according to manufacturers instructions. Cells were then stained for surface markers, washed in PBS with 2% FBS then fixed with CellFix (BDbiosciences) for 20 minutes at 4°C, washed and resuspended in PBS 2% FCS.

Cryopreserved PBMC were thawed into pre-warmed RPMI with 10% FCS and washed and strained through a 40mm cell strainer. Cells were counted using a Countess Automated Cell Counter (Invitrogen) and rested for a maximum of 2 hours. Cells were stained with Zombie UV Fixable Viability Kit (Biolegend) according to manufacturers instructions, followed by surface staining. Cells were washed in FACS buffer (PBS containing 2% FCS and 2 mM EDTA) then fixed and permeabilised for intranuclear staining using Foxp3/Transcription Factor Staining Buffer Set (ThermoFisher).

Instrument

BD LSRFortessa or BD FACSSymphony.

Software

FlowJo v10.10

Cell population abundance

No sorts were performed.

Gating strategy	All gating strategies are shown in the extended data figures.<br><br>Single, live, CD56, CD3 and CD19 negative, low SSC (below the monocyte population) were gated to gate the DC population that were HLA-DR positive CD14 negative.<br><br>Single, live, lymphocytes were gated to gate the NK cell population that were CD56 positive CD3 negative.<br><br>Boundaries between positive and negative were gated either by eye or using the baseline, pre-inocualtion as a background control.

☒ Tick this box to confirm that a figure exemplifying the gating strategy is provided in the Supplementary Information.
